# The Association of Chronic Kidney Disease and Metabolic Syndrome with Incident Cardiovascular Events: Multiethnic Study of Atherosclerosis

**DOI:** 10.1155/2012/806102

**Published:** 2011-07-26

**Authors:** Subhashish Agarwal, Michael G. Shlipak, Holly Kramer, Aditya Jain, David M. Herrington

**Affiliations:** ^1^Cardiology Section, Wake Forest School of Medicine, Winston-Salem, NC 27157, USA; ^2^General Internal Medicine Division, San Francisco VA Medical Center and the University of California San Francisco, San Francisco, CA 94143, USA; ^3^Department of Preventive Medicine and Division of Nephrology and Hypertension, Loyola University Medical Center, Maywood, IL 60153, USA; ^4^Department of Radiology, John Hopkins University, Baltimore, MD 21205, USA

## Abstract

*Background*. There is an association between chronic kidney disease (CKD) and metabolic syndrome (MetS). We examined the joint association of CKD and MetS with incident cardiovascular (CVD) events in the Multiethnic Study of Atherosclerosis (MESA) cohort. *Methods*. We analyzed 2,283 Caucasians, 363 Chinese, 1,449 African-Americans, and 1,068 Hispanics in the MESA cohort. CKD was defined by cystatin C estimated glomerular filtration rate ≤ 60 mL/min/1.73 m^2^ and MetS was defined by NCEP criteria. Cox proportional regression adjusting for age, ethnicity, gender, study site, education, income, smoking, alcohol use, physical activity, and total and LDL cholesterol was performed to assess the joint association of CKD and MetS with incident CVD events. Participants were divided into four groups by presence of CKD and/or MetS and compared to the group without CKD and MetS (CKD^−^/MetS^−^). Tests for additive and multiplicative interactions between CKD and MetS and prediction of incident CVD were performed. *Results*. During follow-up period of 5.5 years, 283 participants developed CVD. Multivariate Cox regression analysis demonstrated that CKD and MetS were independent predictors of CVD (hazard ratio, 2.02 for CKD, and 2.55 for MetS). When participants were compared to the CKD^−^/MetS^−^ group, adjusted HR for the CKD^+^/MetS^+^ group was 5.56 (95% CI 3.72–8.12). There was no multiplicative interaction between CKD and MetS (*P* = 0.2); however, there was presence of additive interaction. The relative excess risk for additive interaction (RERI) was 2.73, *P* = 0.2, and the attributable portion (AP) was 0.49 (0.24–0.74). *Conclusion*. Our findings illustrate that the combination of CKD and MetS is a strong predictor of incident clinical cardiovascular events due to presence of additive interaction between CKD and MetS.

## 1. Introduction

A large percentage of the US population (10%) suffers from chronic kidney disease (CKD) [1] which is associated with metabolic syndrome [2–4]. Metabolic syndrome (MetS) is a construct of physical and laboratory anomalies that confers a higher risk for diabetes mellitus, cardiovascular events and mortality. The National Cholesterol Education Program Adult Treatment Panel (ATP III) criteria define MetS as having at least three of the following: abdominal or central obesity; high triglyceride levels; low high-density lipoprotein (HDL) cholesterol; hyperglycemia; hypertension [5], which has high prevalence in the US [6]. 

Both CKD [7–9] and MetS [10, 11] have been shown to be independently associated with increased cardiovascular events and mortality, and studies suggest that CKD and MetS are associated with each other as well [2–4]. The increased cardiovascular risk of kidney disease is partly explained by an increased burden of traditional cardiovascular risk factors, such as abnormalities in serum lipid concentrations and distribution (elevated triglycerides and lower high-density lipoprotein), diabetes mellitus, and hypertension [2, 12, 13] which are also part of the MetS construct. There is evidence to suggest that CKD progression and associated adverse CVD outcomes are related to severe vitamin D deficiency. The recent findings of vitamin D, being a modulator of both insulin resistance [14] and the renin-angiotensin system [15] and the implication of the renin-angiotensin system in local pancreatic islet structure and function [16, 17], suggest that perhaps renal dysfunction and MetS may share common pathological pathways. This overlap in associated risk factors combined with the potential modifying effect of impaired renal function raises the question about whether the co-occurrence of both conditions would augment or attenuate the anticipated risk based on the effect of the two risk factors individually. The purpose of this study is to understand the joint associations of the two conditions with CVD events in a multiethnic population which could lead to improvements in risk stratification and determine whether participants with both conditions should be specifically targeted for more aggressive and early risk factor interventions. Because cystatin C appears to be more sensitive in detecting mild to moderate decrease in glomerular filtration rate [18, 19], shows strong associations with incident cardiovascular events [20] and all cause mortality [20, 21], and is not affected by age, gender, ethnicity, or muscle mass, this study focused on CKD defined by reduced GFR using a cystatin C-based estimating equation [22]. 

## 2. Materials and Methods

### 2.1. Study Population

The Multiethnic Study of Atherosclerosis (MESA) design has been previously described [23]. Briefly, MESA is a prospective cohort study that began in July 2000 to investigate the prevalence, correlates, and progression of subclinical CVD. The study included 6814 men and women aged 45–84 years old recruited from 6 US communities (Baltimore, MD; Chicago, IL; Forsyth County, NC; Los Angeles County, CA; northern Manhattan, NY; St. Paul, MN). MESA cohort participants were 38% Caucasian (*n* = 2622), 28% African-American (*n* = 1893), 22% Hispanic (*n* = 1496), and 12% Chinese (*n* = 803). Individuals with a history of physician-diagnosed myocardial infarction, angina, heart failure, stroke, or transient ischemic attack, or who had undergone an invasive procedure for CVD (coronary artery bypass graft, angioplasty, valve replacement, pacemaker placement, or other vascular surgeries) were excluded from the study at baseline (2000–2002). After excluding participants with missing data on serum cystatin C (*n* = 58) and covariates (*n* = 1143), we had 5,613 participants at baseline with complete data on serum cystatin C, Mets, and covariates of interest. This study was approved by the Institutional Review Boards of each study site and written informed consent was obtained from all participants.

### 2.2. Laboratory Measures and Data Collection

Medical history, anthropometric measurements, and laboratory data for the present study were taken from the first examination of the MESA cohort (July 2000–August 2002). Information about age, sex, ethnicity, and medical history were obtained by questionnaires. Resting blood pressure was measured using the Dinamap monitor PRO 100 (Critikon, Tampa, Fla, USA) automated oscillometric device. Three measurements were obtained at 1-min intervals with the subject in the seated position with back and arm supported after 5 min of rest with an appropriate-sized cuff, with the cuff at the level of the heart, using a standardized protocol. The average of the second and third measurements was recorded as the resting blood pressure. Hypertension was defined as a systolic blood pressure ≥140 mm Hg, a diastolic blood pressure ≥90 mm Hg, or currently taking medications for blood pressure control [24]. Smoking use was defined as never, former, and current smokers. Smoking ever is defined as ≥100 cigarettes in one's lifetime; current is defined as having smoked a cigarette in the last 30 days. Diabetes was defined as a fasting glucose ≥126 mg/dL or use of insulin or hypoglycemic medications. Plasma lipids (HDL cholesterol, triglycerides, and total cholesterol) were measured from blood samples obtained after a 12-hour fast and measured using a standardized kit (Roche Diagnostics). LDL cholesterol was calculated with the Friedewald equation [25]. Cystatin C was measured from frozen sera at a central laboratory (University of Vermont, Colchester, Vt, USA) using a BNII nephelometer (Dade Behring Inc, Deerfield, Ill, USA) and a particle-enhanced immunonephelometric assay (N Latex Cystatin C; Dade-Behring) [26]. The analytical coefficient of variation for this assay is 2.5%. 

Chronic kidney disease was defined as cystatin C derived glomerular filtration rate ≤60 mL/min/1.73 m^2^ using the formula derived and validated by Stevens et al. [22] (eGFR_cysC_ = 76.7  ∗  cystatin C^−1.19^). The National Cholesterol Education Program/Adult Treatment Panel (NCEP ATP III) [5, 27] definition was used to classify participants having MetS in the MESA cohort. Three of five components are required for diagnosis. (1) Waist circumference ≥102 cm :  men, ≥88 cm : women, (2) hypertension ≥130 mm Hg systolic or ≥85 mm Hg diastolic or use of medications for hypertension, (3) fasting blood glucose ≥100 mg/dL or treatment for impaired fasting glucose, (4) triglycerides ≥150 mg/dL or specific treatment, and (5) HDL-C ≤40 mg/dL in men and ≤50 mg/dL in women.

A dummy variable with four categories for all the possible permutations of CKD and MetS was created. The categories were as follows: (a) no CKD and no MetS (CKD^−^/MetS^−^), (b) CKD and no MetS (CKD^+^/MetS^−^), (c) no CKD and MetS (CKD^−^/MetS^+^), and (d) both CKD and MetS (CKD^+^/MetS^+^).

### 2.3. Cardiovascular Events

A detailed description of events and the process of adjudication can be found at the MESA website (http://www.mesa-nhlbi.org). Briefly, participants were contacted every 9–12 months to inquire about hospital admissions, cardiovascular diagnoses, and deaths. Hospital records were abstracted for possible CVD events and were sent for review and classification by an independent adjudication committee. For the purposes of this study, a CVD event was defined as incident myocardial infarction, resuscitated cardiac arrest, definite angina, probable angina if followed by revascularization, stroke, stroke death, coronary heart disease (CHD) death, other atherosclerotic death, and other CVD death as defined by the MESA protocol.

### 2.4. Statistical Analysis

 Descriptive analyses of all the variables utilized in the data analysis were conducted. The baseline features were compared, using ANOVA or Kruskal-Wallis tests for continuous variables and the chi-square or the Fisher exact tests for categorical variables, into four columns: Neither CKD/MetS; CKD only; MetS only; both CKD and MetS. 

A Cox proportional hazards regression with and without adjustment for age, ethnicity, gender, study site, education, income, smoking, alcohol use, physical activity, total, and LDL cholesterol was performed to assess the independent association of CKD and MetS with incident CVD events with CKD and MetS in the same model. 

We next divided the participants into four groups according to the presence/absence of CKD and/or MetS. Survival analysis was performed using cumulative event-free Kaplan-Meier curves according to the presence/absence of CKD or MetS, and the groups were compared by log-rank test for trend. A similar Cox proportional hazards regression was performed to investigate the relationship of the four groups (neither CKD/MetS, CKD only, MetS only, nor both CKD and MetS) with incident CVD events using two sets of models: unadjusted models and models adjusted (Table 2) for established cardiovascular risk factors (age, ethnicity, gender, study site, education, income, smoking, alcohol use, physical activity, and total and LDL cholesterol) using CKD^−^/MetS^−^ as the reference category. The potential confounders were selected based on their relationship with cardiovascular disease and the prior literature. Additionally, a similar analysis was performed after excluding participants with prevalent diabetes.

Formal tests for additive and multiplicative interactions between CKD and MetS and prediction of incident CVD were also performed. We tested additive and multiplicative interactions in the proportional hazards model. A formal interaction term CKD × MetS was introduced in the model with all covariates to test for multiplicative interaction.

Formal tests for indices of additive interaction such as relative excess risk due to interaction (RERI), attributable portion (AP), and synergy index (SI) were performed as described by Li and Chambless [28]. RERI is calculated as ((HR (both CKD and MetS) − HR (CKD alone) − HR (MetS alone) + 1)). AP is calculated as RERI divided by HR (both CKD and MetS). SI is ratio of increase in hazard due to both exposures (CKD and MetS) to the sum of the increases due to one exposure alone. Please see The appendix for formal calculations. All statistical analyses were performed using JMP Version 8 (SAS Institute Inc., Cary, NC/USA).

## 3. Results

### 3.1. Participant Characteristics

The sociodemographic characteristics of the study sample are depicted in Table 1. The mean age of the sample was 61.6 years with the mean age being much higher at 69.7 years for participants with both CKD and MetS. A larger proportion of Chinese-Americans had no CKD/MetS (81%) compared to other ethnicities, and only 1% of Chinese-Americans had both the conditions compared to 3% for all other ethnicities. Among women, 31% had MetS as compared to 26% of men. Diabetes was more prevalent in the groups with MetS and both CKD/MetS compared to those without MetS or CKD/MetS. Smoking rates were not much different between the groups, whereas alcohol consumption was significantly higher in healthy participants. Additionally, the healthy participants were significantly more physically active as compared to participants with either or both conditions.

### 3.2. Cardiovascular Events

**Figure 1:**
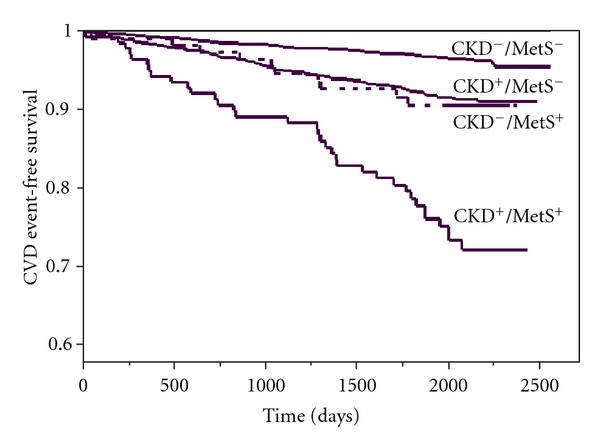
Kaplan-Meier plots showing cumulative CVD event-free survival in participants in four groups divided by presence or absence of CKD and presence or absence of MetS (log-rank test for trend *X* ^2^ = 114; *P* < 0.001); CKD: chronic kidney disease; MetS: metabolic syndrome; CVD: cardiovascular disease.

During 5.5 years of followup, 283 CVD events were identified (118, CKD^−^/MetS^−^ group; 10, CKD^+^/MetS^−^ group; 120, CKD^−^/MetS^+^ group; 35, CKD^+^/MetS^+^ group). A Kaplan-Meier survival curve shows decreased survival free of CVD events across the four groups of CKD and MetS with a log test for trend which is statistically significant (*P* < 0.0001) (Figure 1). These curves show significantly poorer survival in the CKD^+^/MetS^+^ group.

When CKD and MetS were entered into the same model, the results of multivariate Cox regression analysis including age, sex, ethnicity, smoking habit, cholesterol both total and LDL-C, alcohol consumption, and physical activity found that CKD (HR 2.02, 95% CI 1.43–2.79, *P* < 0.0001) and MetS (HR 2.55, 95% CI 2.01–3.25, *P* < 0.0001) were both significantly associated with incident CVD events.

Table 2 shows the results from a series of crude and multivariate regression analysis, showing how the association of CKD and MetS with CVD risk changed as groups of CVD risk factors were added to the regression model. In the crude model, the risk for CVD was significantly higher in the CKD^+^/MetS^+^ group compared with the MetS^−^/CKD^−^ group (HR 8.46). The hazard in the CKD^+^/MetS^+^ group remained highly significant in the multivariate model (HR 5.56). It remained significant even after further adjustment for antihypertensive medications and systolic blood pressure (HR 4.55, 95% CI 3.01–6.73). Furthermore, when compared with the CKD^+^/MetS^−^ group or with the CKD^−^/MetS^+^ group, the risk of CVD events was significantly higher in the CKD^+^/MetS^+^ group in univariate Cox regression analysis (versus CKD^+^/MetS^−^ group: HR 3.14, 95% CI 1.61–6.69, *P* < 0.001; versus CKD^−^/MetS^+^ group: HR 3.38, 95% CI 2.28–4.87, *P* = 0.0001) and in multivariate Cox regression analysis (versus CKD^+^/MetS^−^ group: HR 3.89, 95% CI 1.99–8.31; versus CKD^−^/MetS^+^ group: HR 2.32, 95% CI 1.56–3.37, *P* < 0.0001, resp.).

We performed several additional analyses to address the robustness of these findings. Because patients with diabetes were more frequent in the CKD^+^/MetS^+^ group, we repeated our analysis for the 4,591 participants without previous diabetes. In this study, 210 CVD events occurred during the follow-up period. The independent predictive value of CKD^+^/MetS^+^ for CVD events was also confirmed by the Kaplan-Meier method (log rank test for trend chi-square = 60; *P* < 0.0001) and by multivariate Cox regression analysis. Furthermore, even when compared with the CKD^+^/MetS^−^ group or with the CKD^−^/MetS^+^ group, the risk of CVD events was significantly higher in the CKD^+^/MetS^+^ group in the multivariate model (versus CKD^+^/MetS^−^ group: HR 3.13, 95% CI 1.48–7.03, *P* = 0.03; versus CKD^−^/MetS^+^ group: HR 2.13, 95% CI 1.23–3.49, *P* = 0.01).

Finally, when interaction was tested between CKD and MetS, no multiplicative interaction was demonstrated (CKD × MetS, *P* = 0.2). When formal tests for additive interaction such as relative excess risk due to interaction (RERI), attributable portion (AP), and synergy index (SI) were performed, there was presence of significant additive interaction as shown in Table 3 and Appendix. RERI (95% CI) was estimated at 2.73 (0.57–4.85, *P* = 0.02), AP (95% CI) was estimated at 0.49 (0.24–0.74), and SI (95% CI) was estimated at 2.49 (1.24–4.98). According to the three measures of additive interaction between CKD and MetS, there is 2.73 relative excess risk due to the additive interaction, the risk of CVD in individuals who had been exposed to both risk factors (CKD and MetS) is 2.49 times higher than the sum of risks in individuals exposed to a single risk factor alone, and 49% of the incident CVD in individuals exposed to both risk factors is attributable to the additive interaction.

## 4. Discussion

In this ethnically diverse population of 5,163 individuals, aged 44–84, both chronic kidney disease and metabolic syndrome are independent predictors of incident cardiovascular events. This study identified a significant positive relationship between the cooccurrence of CKD and MetS and risk for CVD events. In a multivariate model, the hazard for incident cardiovascular events was increased due to presence of significant additive interaction between CKD and MetS. However, no multiplicative interaction between CKD and MetS was demonstrated. Additionally, the presence of both CKD and MetS conferred a significantly higher hazard compared to the presence of each condition separately. From the viewpoint of prevention and clinical practice, additive interaction is more important than multiplicative interaction, as it relates to a higher absolute excess of cases.

Defined by cystatin C, CKD [7, 29] has been shown to predict cardiovascular events and mortality in several studies. Ix et al. [7] studied 990 participants in the Heart and Soul Study and demonstrated that compared to participants in the lowest cystatin C quartile, those in the highest quartile (those with CKD) were at increased risk of cardiovascular events (HR, 2.0; 95% CI, 1.0–3.8). Similarly, Deo et al. [29] studied 3,044 older adults ages from 70 to 79 over 6 years in the Health ABC cohort and found that those with CKD had significantly higher risk for cardiovascular death (HR, 2.24; 95% CI, 1.30–3.86) compared to those without CKD. This is similar to our findings where participants with CKD, as defined by eGFR_cysC_ ≤ 60 mL/min/1.73 m^2^ were at increased risk for CVD events (HR, 2.02, 95% CI, 1.43–2.79) in a multivariate analysis. However, when the CKD only group was compared to participants with no CKD and no MetS, the hazard for CVD events was statistically insignificant, which is a reflection of low statistical power (Table 2). 

Similarly, MetS has been shown to predict CVD events and mortality [11]. A meta-analysis involving 43 cohorts consisting of 172,573 individuals showed that MetS had a relative risk of cardiovascular events and death of 1.78 (95% CI, 1.58–2.00). In our study, presence of MetS was an independent predictor of CVD events, and when the MetS only group was compared to the group with no CKD/no MetS, the hazard for CVD events remained statistically significant (Table 2).

Multiple studies [2–4] document the associations between CKD and MetS, and now mechanisms have been postulated that link the two conditions to each other. Recent findings, suggesting vitamin D being a modulator of both insulin resistance and the renin-angiotensin system [15] and the renin-angiotensin system in local pancreatic islet structure and function [16], suggest that perhaps renal dysfunction and MetS may share common pathological pathways. Clinically, it is seen that individuals with CKD have abnormalities in serum lipid concentrations and distribution (elevated triglycerides and lower high-density lipoprotein), diabetes mellitus, and hypertension [2, 12, 13] which are also part of the MetS construct. Additionally, it has been proposed that the presence of both conditions leads to increased inflammation and oxidative stress, increased total peripheral resistance, and impaired left ventricular relaxation which increases the risk for CVD events [30]. This interplay of risk factors and pathological mechanisms implies that perhaps the cooccurrence of CKD and MetS identifies a group of individuals at higher risk for cardiovascular events. 

A few studies [30, 31] document the role of these two conditions together as it relates to CVD events. Martin et al. [31] studied 13,115 individuals aged ≥35 years from the NHANES III survey and found that the coclustering of CKD and MetS led to a significantly higher hazard for CVD mortality (HR, 3.23; 95% CI, 2.56–3.70) when compared with individuals with no CKD and no MetS. Similarly, Iwashima et al. [30] studied 1,160 essential hypertensive individuals for a mean period of 4.8 years and found that the presence of both CKD and MetS conferred a higher risk for CVD events (HR, 3.58; 95% CI, 2.14–5.95) compared to the no CKD/no MetS group. Our findings are a validation and extension of these findings in a multiethnic cohort free of cardiovascular disease at baseline. In contrast to the study by Martins, we found no significant association in the CKD only group but found significant association in the MetS only group which is perhaps due to small numbers of CKD only individuals. Second, our study included a cohort with both with and without hypertension.

Our study has several limitations. First, MESA did not directly measure glomerular filtration rate (GFR); therefore, we cannot be certain that the association between elevated cystatin C level and CVD events are solely caused by its approximation of impaired GFR. This approach can lead to misclassification of individuals due to biased estimates. Second, although efforts were made to adjust for known confounders, there remains a possibility of failure to adjust for unknown confounders or inadequate adjustment of established risk factors (severity and duration of hypertension, diabetes) resulting in spurious results due to residual confounding. Third, some studies suggest that corticosteroids [32] and thyroid function [33] are associated with cystatin C, and since adjustment with these measures was not performed, results should be interpreted with caution in this subset of individuals. The distribution of metabolic syndrome components may vary from population to population, which may have an impact on the external validity of findings if the joint association/interaction is mostly due to one of the components. The results of the study are limited to individuals without cardiovascular disease and may not be generalizable to a population with known coronary artery disease due to selection bias. Also, due to the cross-sectional nature of the risk factors, the association between CKD, MetS, and CVD events could potentially be due to post assessment residual confounding.

## 5. Conclusion

This study shows that the co-occurrence of CKD and MetS results in an increased hazard for cardiovascular events in a multiethnic population. Although, no multiplicative interaction was demonstrated, there is significant presence of additive interaction. Both CKD and MetS are independent predictors of CVD but their combination furthers the risk independent of conventional risk factors. From the clinical viewpoint, physicians should become more cognizant that concomitant CKD and MetS lead to increased risk for CVD events. Additionally, assessment of renal function in individuals with MetS and vice versa may lead to improved risk stratification for cardiovascular disease in clinical practice. More studies are needed in the future to explore the temporal relationship between CKD, MetS and cardiovascular disease. Additionally, studies are needed to explore whether novel and aggressive pharmacological and behavioral modifications in individuals with both CKD and MetS, will lead to reduction in CVD risk.

##  Disclosures

The authors had full access to the data and take responsibility for the integrity of the data. All authors have read and agree to the study as written. 

## Figures and Tables

**Table 1:** Baseline characteristics of study participants in the MESA cohort at baseline (2000).

Variables	Total	CKD^−^/MetS^−^	CKD^+^/MetS^−^	CKD^−^/MetS^+^	CKD^+^/MetS^+^	*P* value
*n*	5163	3444	119	1455	145	
Age, years	61.6 (10.1)	60.7 (10.1)	71.1 (9.7)	62.2 (9.6)	69.7 (9.3)	0.0001
Caucasian	2283 (44%)	1565(69%)	65 (3%)	581 (25%)	72 (3%)	0.0002
Chinese	363 (7%)	293 (81%)	5 (1%)	61 (17%)	4 (1%)	0.0001
African	1449 (28%)	953 (66%)	29 (2%)	428 (30%)	39 (3%)	0.0001
Hispanic	1068 (21%)	633 (59%)	20 (2%)	385 (36%)	30 (3%)	0.0001
Male, %	2722 (53%)	1872(69%)	74 (3%)	697 (26%)	79 (3%)	0.0001
Female, %	2441 (47%)	1572 (64%)	45 (2%)	758 (31%)	66 (3%)	0.0001
DM, %	572 (11%)	128 (4%)	2 (2%)	394 (27%)	48 (33%)	0.0001
Current smokers, %	741 (14%)	491 (14%)	19 (16%)	213 (15%)	18 (12%)	0.8
Current drinking, %	3576 (69%)	2494 (72%)	78 (66%)	919 (63%)	85 (59%)	0.0001
Physical activity, min/wk	1644 (2395)	1777 (2537)	1670 (2855)	1372 (2012)	1205 (1683)	0.0001
Total cholesterol, mg/dL	193 (35)	194 (34)	187 (38)	193 (36)	188 (39)	0.08
SBP, mmHg	126 (21)	122 (20)	130 (23)	133 (21)	137 (25)	0.0001
DBP, mmHg	72 (10)	72 (10)	71 (10)	74 (10)	72 (11)	0.0001
LDL-C, mg/dL	117 (31)	118 (31)	113 (32)	116 (33)	113 (31)	0.01
HDL-C, mg/dL	51 (15)	55 (15)	52 (14)	43 (10)	42 (11)	0.0001
TG, mg/dL	125 (65)	103 (49)	109 (43)	173 (72)	170 (67)	0.0001
Fasting glucose, mmHg	96 (27)	90 (19)	89 (9)	110 (40)	109 (33)	0.0001
eGFR_cysC_, mL/min/1.73m^2^	93 (22)	98 (20)	51 (9)	90 (19)	49 (10)	0.0001
MetS components, %						
HTN, %	2219 (43%)	1078 (31%)	70 (59%)	954 (66%)	117 (81%)	0.0001
Obesity, %	2763 (54%)	1319 (38%)	57 (48%)	1259 (87%)	128 (88%)	0.0001
Elevated TG, %	1457 (28%)	415 (12%)	13 (11%)	939 (66%)	90 (62%)	0.0001
Low HDL-C, %	1957 (38%)	736 (21%)	32 (27%)	1078 (74%)	111 (77%)	0.0001
Impaired Glucose, %	2062 (40%)	1037 (30%)	39 (33%)	892 (61%)	94 (65%)	0.0001
CVD events, %	283 (5%)	118 (3%)	10 (8%)	120 (8%)	35 (24%)	0.0001

*P* values obtained by one aay analysis of variance. Data presented in total numbers (percentages) and continuous measures presented as mean value (standard deviation). DM: diabetes mellitus; SBP: dystolic blood pressure, mmHg; DBP: diastolic blood pressure, mmHg; LDL-C: mg/dL low density lipoprotein cholesterol; HDL-C: mg/dL high density lipoprotein cholesterol; TG: mg/dL, triglyceride; eGFR_cysC_: mL/min/1.73 m^2^ glomerular filtration rate estimated from cystatin C; CVD: cardiovascular events; MetS: metabolic syndrome; CKD: chronic kidney disease.

**Table 2:** Unadjusted and multivariate-adjusted HRs of CVD events associated with CKD and MetS.

	Total participants (*n* = 5,163)		Participants without diabetes (*n* = 4,591)	
	Crude	Adjusted	Crude	Adjusted
CKD^−^/MetS^−^	1 (Reference)	1 (Reference)	1 (Reference)	1 (Reference)

CKD^+^/MetS^−^	2.70 (1.32−4.88)	1.43 (0.70−2.62)	2.90 (1.42−5.26)	1.41 (0.69−2.61)

CKD^−^/MetS^+^	2.51 (1.94−3.23)	2.40 (1.85−3.11)	2.17 (1.60−2.91)	2.08 (1.54−2.81)

CKD^+^/MetS^+^	8.46 (5.72−12.20)	5.56 (3.72−8.12)	7.27 (4.33−11.54)	4.43 (2.60−7.15)

Hazard ratios (95% CI) adjusted for age, ethnicity, gender, study site, education, income, smoking, alcohol use, physical activity, and total and LDL cholesterol. HR: hazard ratio; CI: confidence interval; CVD: cardiovascular events; CKD: chronic kidney disease; MetS: metabolic syndrome.

**Table 3:** Estimates of multiplicative and additive interaction (95% confidence interval, CI) controlling for covariates.

	CKD × MetS		
Parameters	Estimate	95% CI	*P*-value
*β*_3_	0.48	−0.23, 1.28	0.2
RERI	2.73	0.57, 4.85	0.02
AP	0.49	0.24, 0.74	
SI	2.49	1.24, 4.98	

CKD: chronic kidney disease; MetS: metabolic syndrome; *β*_3_: parameter estimate of CKD × MetS testing for multiplicative interaction; RERI: relative excess risk due to additive interaction; AP: attributable portion; SI: synergy index are measures testing for additive interaction.

**Table 4:** Output from proportional hazards models.

	Parameter	Estimated *β*	SE (*β*)	*t*-test	*P* value	HR	95% CI
	CKD	0.36	0.33	1.14	0.29	1.43	0.74−2.75
Model*	MetS	0.87	0.13	44.05	0.0001	2.40	1.85−3.10
	CKD × MetS	0.48	0.38	1.61	0.2	1.62	0.77−3.44

*Parameter estimates and test statistics for interaction between CKD (chronic kidney disease) and MetS (metabolic syndrome) adjusted for covariates.

**Table 5:** Covariance matrix of the set of *β* coefficients from the proportional hazards models.

	*β*_1_ (CKD)	*β*_2_ (MetS)	*β*_3_ (CKD × MetS)
*β*_1_(CKD)	0.1119220903	0.0087980044	−0.1097780898
*β*_2_(MetS)	0.0087980044	0.0173595635	−0.0170957203
*β*_3_(CKD × MetS)	−0.1097780898	−0.0170957203	0.1463599444

## Appendix

Using the output from Tables 4 and 5, the calculations of RERI and the test for the additive interaction are illustrated below.

(1) Relative Excess Risk due to Interaction (RERI) = HR_11_ − HR_10_ − HR_01_ + 1, where HR_11 _is hazard due to both CKD and MetS, HR_10_ is hazard due to CKD alone, and HR_01 _is hazard due to MetS alone.

(A.1) $$\begin{array}{cc} \text{RERI}\; & ={\text{e}}^{\;\beta 1+\;\beta 2+\;\beta 3}-{\text{e}}^{\beta 1}-{\text{e}}^{\beta 2}+1\\ & =5.56-1.43-2.40+1=2.73,\\ \text{VAR}\left(\text{RERI}\right) & =a_{2}^{1}\times \text{var}\beta_{1}+a_{2}^{2}\times \text{var}\beta_{2}+a_{3}^{2}\times \text{var}\beta_{3}\\ & \;+2\;(a_{1}\;a_{2}\times \text{Cov}\left(\beta_{1}\;\beta_{2}\right)+a_{1}a_{3}\\ & \;\times \text{Cov}\left(\beta_{1}\beta_{3}\right)+a_{2}a_{3}\times \text{Cov}\left(\beta_{2}\beta_{3}\right)),\\ a_{1} & ={\text{e}}^{\beta 1+\beta 2+\beta 3}-{\text{e}}^{\;\beta 1}\\ & =5.56-1.43=4.13,\\ a_{2} & ={\text{e}}^{\beta 1+\beta 2+\beta 3}-{\text{e}}^{\beta 2}\\ & =5.56-2.40=3.16,\\ a_{3} & ={\text{e}}^{\beta 1+\beta 2+\beta 3}\;=5.56. \end{array}$$

VAR (RERI) = 1.19, SE (RERI) = 1.09. Hence, *t* = RERI/VAR (RERI) = 2.73/1.19 = 2.28, *P* = 0.02, and a 95% confidence interval estimate for RERI, RERI ±  1.96 × SE (RERI) = (0.57 − 4.85):

(2) Attributable portion (AP) = (e^*β*1+*β*2+*β*3^ − e^*β*1^ − *e* ^*β*2^ + 1)/(e^*β*1+*β*2+*β*3^) = 2.73/5.56 = 0.49:

(A.2) $$\begin{array}{c} a_{1}=\frac{{\text{e}}^{\beta 2-1}}{{\text{e}}^{\beta 1+\beta 2+\beta 3}},\\ a_{2}=\frac{{\text{e}}^{\;\beta 1-1}}{{\text{e}}^{\beta 1+\beta 2+\beta 3}},\\ a_{3}=\frac{{\text{e}}^{\;\beta 1+\beta 2-1}}{{\text{e}}^{\beta 1+\beta 2+\beta 3}}. \end{array}$$

VAR(AP) = 0.016, SE(AP) = 0.126. Hence, 95% confidence interval estimate for AP, AP ± 1.96 × SE(AP) = (0.24 − 0.74).

(3) Synergy index (SI) = e^*β*1+*β*2+*β*3^ − 1/e^*β*1+*β*2^ − 2 = 4.56/1.83 = 2.49.

(A.3) $$\begin{array}{c} a_{1}=a_{3}-\left(\frac{{\text{e}}^{\;\beta 1}}{{\text{e}}^{\beta 1+\beta 2}}-2\right),\\ a_{2}=a_{3}-\left(\frac{{\text{e}}^{\beta 2}}{{\text{e}}^{\beta 1+\beta 2}}-2\right),\\ a_{3}=\left(\frac{{\text{e}}^{\beta 1+\beta 2+\;\beta 3}}{{\text{e}}^{\beta 1+\beta 2+\beta 3}}-1\right). \end{array}$$

VAR(log S) = 0.125, SE(Log S) = 0.354. Hence, 95% confidence interval estimate for SI, SI ± 1.96 × SE(SI) = (1.24 − 4.98).
